# Improvement in Analytical Methods for Determination of Sugars in Fermented Alcoholic Beverages

**DOI:** 10.1155/2018/4010298

**Published:** 2018-10-08

**Authors:** Ayalew Debebe, Shibru Temesgen, Mesfin Redi-Abshiro, Bhagwan Singh Chandravanshi, Estifanos Ele

**Affiliations:** ^1^Department of Chemistry, Addis Ababa University, P.O. Box 1176, Addis Ababa, Ethiopia; ^2^Department of Chemistry, Haramaya University, P.O. Box 138, Dire Dawa, Ethiopia; ^3^Department of Statistics, Addis Ababa University, P.O. Box 1176, Addis Ababa, Ethiopia

## Abstract

The main objective of this study was to improve the performance of analytical methods for the determination of sugars in fermented alcoholic beverages based on mid-infrared-partial least squares (MIR-PLS), high-performance liquid chromatography with the ultraviolet detector (HPLC-UV), high-performance liquid chromatography with the refractive index detector (HPLC-RI), and sulfuric acid methods. The MIR-PLS method was found to give good prediction of individual sugars: glucose, fructose, sucrose, and maltose in the alcoholic beverages with less than 4% error. The HPLC-UV method can be used for the determination of glucose in alcoholic beverages after derivatization with *p*-aminobenzoic acid ethyl ester. The HPLC-RI method was found to be applicable for the determination of individual sugars: glucose, fructose, sucrose, and maltose in the alcoholic beverages. The limit of detection (%, w/w) and recovery (%) of the individual sugars by the HPLC-RI method were fructose 0.001, 89.4–106; glucose 0.002, 92.4–109; and sucrose 0.002, 94.2–95.1. The sulfuric acid method was found to be useful for the determination of total sugar in the alcoholic beverages. The limit of detection (%, w/w) and recovery (%) of the total sugar by the sulfuric acid method were found to be 0.009, 98.2–109. The HPLC-RI method was applied to determine the level of individual sugars, while the sulfuric acid method was used to determine total sugar in Ethiopian traditional fermented alcoholic beverages: *Tella*, *Netch Tella*, *Filter Tella*, *Borde*, *Tej*, *Korefe*, *Keribo*, and *Birz*. The sugar contents in the real samples were found in the ranges (%): glucose 0.07–5.60, fructose 0.09–8.50, sucrose and maltose 0.08–3.00, and total sugar 12.0–64.5. The levels of sugars in Ethiopian traditional fermented alcoholic beverages were found to be comparable with literature data.

## 1. Introduction

Carbohydrates are one of the most abundant compounds in foods [1–6]. They are classified into five major classes, which are monosaccharides, disaccharides, oligosaccharides, polysaccharides, and nucleotides [7]. Mono- and disaccharides with a sweet flavor are commonly called sugars [8].

Alcoholic beverages contain sugars and other soluble solids [9]. In beverages, especially in beer, 80–85% of nonvolatile materials (30–40 g/L) are sugars [10]. Fructose, glucose, maltose, sucrose, and maltotriose (but not lactose) are fermentable sugars [10]. However, oligosaccharides containing more than three glucose units cannot be fermented [10]. Thus, when fermentation is completed, only small amounts of lower sugars are found in alcoholic beverages [10].

The determination of sugars in alcoholic beverages has been reported using a large number of analytical techniques such as chromatographic techniques, spectroscopic techniques, colorimetric methods, iodometric methods, and enzymatic techniques [11]. Iodometric methods [11] and traditional colorimetric methods [12] are unable to quantitate sugars individually. Chromatography and capillary electrophoresis methods require derivatization (indirect method) because carbohydrates are nonvolatile [1, 13] and lack both charge and strong chromophore [11]. Although derivatization provides high sensitivity, it is complicated and time-consuming [11]. Both gas and liquid chromatographic techniques have used different derivatizing reagents. For gas chromatography, trimethylsilyl (TMS) ethers are the most popular derivatizing agent [14]. However, TMS derivatization has limitations such as it is unstable, forms multiple peaks, and has difficulty in preparation [13, 14]. Likewise, among different UV active and fluorophore-derivatizing agents, *p*-aminobenzoic acid ethyl ester (ABEE) is the most widely known one [1]. The derivatization of reducing sugars with ABEE is easy and required no special equipment; moreover, the method showed higher sensitivity and elimination of the possible doublet that could be formed by mutarotation of the free reducing end of sugars [1]. In the direct method, high-performance liquid chromatography (HPLC) has been used with pulsed amperometric, evaporative light-scattering, and refractive index detectors [1, 15] and ultraviolet detectors at 195 nm [3]. A direct method with the sugar-borate complex using capillary zone electrophoresis (CZE) has been used. However, it takes about 20 min to analyze several carbohydrates because the sugar-borate complexes migrate against the direction of the EOF [11]. Chromatographic methods have high accuracy, but they are often time-consuming, labor intensive, and require tedious and complex processing. Recently, near-infrared (NIR) and middle-infrared (MIR) spectrometries have become popular. They have been applied in a low-cost, rapid, and nondestructive way [16]. However, different techniques have their own advantages and disadvantages.

The determination of sugars in alcoholic beverages has considerable importance [1–4, 17, 18]. It is useful to know the contribution of sugars to flavor and sensory characteristics to evaluate their nutritive (caloric) value [1], to evaluate how they are formed and assimilated [10], and to know their health impacts [19].

The literature survey revealed that only a few studies have been conducted on the determination of chemical composition of Ethiopian traditional fermented beverages. These include determination of phenolics [20], alcohol contents [21–23], and minerals [24]. However, no study has been reported on the sugar contents of traditional fermented beverages.

Therefore, the objectives of this study are (i) to develop new analytical methods for the determination of sugars in fermented alcoholic beverages based on mid-infrared-partial least squares (MIR-PLS), high-performance liquid chromatography with the refractive index detector (HPLC-RI), high-performance liquid chromatography with the ultraviolet detector (HPLC-UV), and sulfuric acid methods, (ii) to compare the analytical parameters of the newly developed methods, and (iii) to determine the level of sugars in Ethiopian traditional fermented alcoholic beverages: *Tella*, *Netch Tella*, *Filter Tella*, *Borde*, *Tej*, *Korefe*, *Keribo*, and *Birz*.

## 2. Materials and Methods

### 2.1. Instrumentation

UV-Vis spectrophotometer (Lambda 950; PerkinElmer, UK) with a 1 cm path length quartz cuvette was used to determine total carbohydrates. The Fourier-transform infrared spectrometer (Spectra 65; PerkinElmer, UK) with ZnSe window as a sample holder, and HPLC-UV and HPLC-RI (Agilent Technologies, Germany) were used for the determination of individual sugars.

### 2.2. Reagents and Chemicals

Ethanol (99.99%; Fisher Scientific, UK), glucose (laboratory reagent; Merck Extra Pure, England), fructose (laboratory reagent; Pharmacos Ltd, England), sucrose (analytical reagent; Guangdong Guanghua Chemical Factory Co. Ltd, China), and maltose (laboratory reagent; The British Drug Houses Ltd, Poole, England) were used to prepare synthetic calibration and validation sets. *p*-Amino benzoic acid ethyl ester (ABEE) (Riedel-de Haen AG, Seelze, Hanover, Germany), acetic acid (99.5%; BDH Chemicals Ltd, Poole, England), and sodium borohydride (BDH Chemicals Ltd, Poole, England) were used for derivatizing sugars. Sulfuric acid (Sigma-Aldrich, Germany) was used for the determination of total carbohydrate. Chloroform (Carlo Erba Reagents, France) was used for purifying the derivatized compound. Distilled deionized water was used for the preparation of standards and dilution of samples.

### 2.3. Preparation of Standard Solutions

Calibration and validation sets were prepared by mixing ethanol with sugars for sugar determination with ethanol standards by the MIR-PLS method. The compositions of the standards were ethanol (2–12%, w/w), fructose (0–5%, w/w), glucose (0–5%, w/w), sucrose (0–5%, w/w), and maltose (0–5%, w/w), whereas for sugar standards without ethanol, the compositions of the standards were glucose (0–14.2%, w/w), fructose (0–17.3%, w/w), sucrose (0–23.4%, w/w), and maltose (0–15.01%, w/w).

For the HPLC-RI method, average calibration curves were developed with standard solutions of glucose, fructose, and sucrose from 0.03% (w/w) to 0.2% (w/w).

For the HPLC-UV method, the average calibration curve was constructed with standard solutions of glucose from 0.0002 to 0.002 mg/L which were derivatized using ABEE.

For the sulfuric acid method, the average calibration curve was constructed with the series of glucose standard solutions in the range 0.01–0.1 g/L.

### 2.4. Sampling and Sample Preparation

Eight most popular Ethiopian traditional fermented beverages, *Tej* (honey wine), *Tella* (a malt beverage like beer), *Korefe*, *Keribo*, *Birz*, *Netch Tella*, *Filter Tella*, and *Borde*, were selected for this study. A total of 57 samples: 15 *Tej*, 15 *Tella*, 6 *Korefe*, 6 *Keribo*, 4 *Birz*, 4 *Netch Tella*, 4 *Filter Tella*, and 3 *Borde*, were collected randomly from vending houses at different subcities of Addis Ababa (Ethiopia) and nearby towns (Sebeta, Dukem, Sululta, Sendafa, and Burayu) of Oromia Regional State. The characteristics of alcoholic samples (the pH value and ethanol content) and brief information about raw materials and processes used for the production of Ethiopian traditional fermented beverages are given in Table 1. A 500 mL aliquot of each type of the beverages was collected from the three sites of each of the subcities of Addis Ababa and nearby towns. A 1000 mL bulk sample was prepared for each sample type from one specific sampling site. This was done by taking 333.3 mL of the beverage from each of the three samples from one place and mixing well in a 1 L volumetric flask. All the samples were collected using glass amber bottles and kept at 4°C until the analysis time. Ethiopian traditional fermented alcoholic beverages are either liquid or semiliquid. The liquid samples such as *Tella*, *Tej*, *Birz*, *Netch Tella*, *Filter Tella*, and *Keribo* were filtered before analysis. The sugars were extracted from the semiliquid samples such as *Borde* and *Korefe* by an optimized procedure.

### 2.5. Derivatization of Sugar for the HPLC-UV Method

The derivatization procedures of sugar with ABEE reported by Gomis et al. [1] and Munegumi and Goto [31] were different. Thus, a modified procedure was used in this study. An ABEE methanol solution (4 mL, 0.5 g/mL) and glacial acetic acid (310 mL) were dissolved at 40–50°C in a polypropylene tube. A 0.60 g sodium borohydride was added to the tube, which was sealed with a screw cap and vortexed to give an ABEE stock solution. An aliquot (2 mL) of the ABEE stock solution and a standard saccharide solution or sample (500 *µ*L) were mixed by vortexing, and the resulting solution was heated at 80°C for 6 h. After cooling and centrifuging for 1 min, the filtrate was treated with 3 mL water and centrifuged. Again, the filtrate was treated with chloroform (2 × 5 mL). Finally, the upper layer (the aqueous phase) was used for HPLC analysis after filtration using a micromembrane (0.45 mm pore size).

### 2.6. Procedure for Total Sugars

A 1 mL aliquot of carbohydrate solution was rapidly mixed with 3 mL of concentrated sulfuric acid in a test tube and vortexed for 30 s. The temperature of the mixture was raised rapidly within 10–15 s after addition of sulfuric acid. The solution was cooled in ice for 2 min to bring it to room temperature. Finally, UV light absorption at 315 nm was measured using a UV spectrophotometer. Reference (reagent blank) solutions were prepared following the same procedure as above, except that the carbohydrate aliquot was replaced with distilled deionized water [12].

### 2.7. HPLC Conditions for Indirect (Derivatized) Determination

The derivatized sample was analyzed to determine the sugars using HPLC with the UV detector at *λ*_max_ 230 nm. The chromatographic separation was achieved on a C_18_ column maintained at 45°C. A binary solvent system comprising 0.5% aqueous trifluoroacetic acid as solvent A and acetonitrile as solvent B was used under the gradient mode. The gradient condition was 0–5 min solvent A, 5–10 min 0–20% B, 10–35 min 20–25% B, 35–50 min 25–45% B, and 50–60 min 45–100% B. The mobile-phase flow rate was 0.5 mL·min^−1^, and sample injection volume was 3 *µ*L.

### 2.8. HPLC Conditions for Direct (Nonderivatized) Determination

The sugar determination was done using HPLC with the RI detector. The chromatographic separation was achieved in the Hi-Plex H column (7.7 × 300 mm) maintained at 35°C. The solvent used was distilled-deionized water. The mobile-phase flow rate and sample injection volumes were 0.5 mL·min^−1^ and 10 *µ*L, respectively.

## 3. Results and Discussion

### 3.1. Extraction of Sugars

For the extraction of sugars from the semiliquid samples, the types and amounts of the extracting solvent and extraction time were optimized. 80% (v/v) methanol, 80% (v/v) ethanol, and water were considered as the extracting solvent. Among the solvents checked, 80% (v/v) methanol showed better efficiency than others (the data are not presented). The extraction time was varied in the range 30–120 min. The optimum time for extraction of sugars was found to be 90 min. Hence, 90 min was used to extract sugars from the beverages (Table 2).

### 3.2. Determination of Individual Sugars Using MIR-PLS

**Figure 1:**
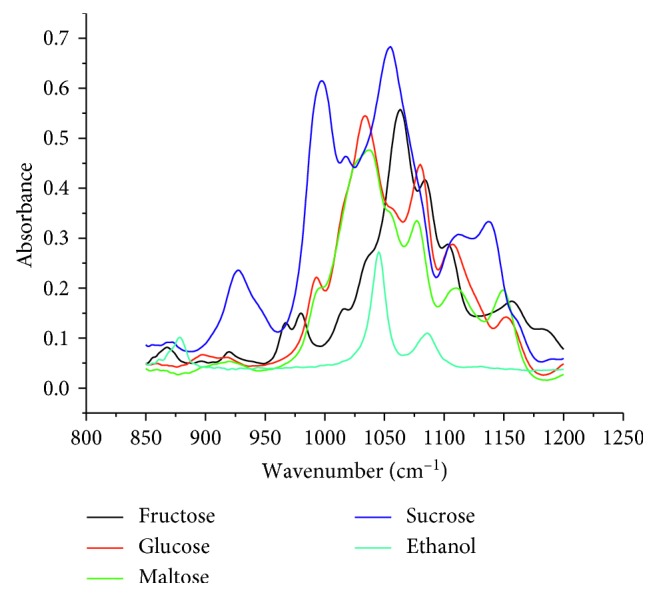
MIR spectra of 5% (w/w) ethanol and 10% (w/w) individual sugars.

**Figure 2:**
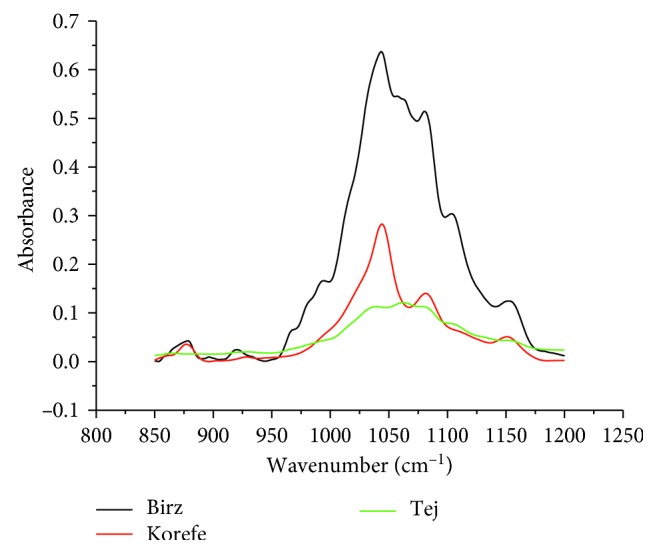
MIR spectra of selected fermented alcoholic beverages.

Carbohydrates have strong absorption bands which overlap in the spectral region, 850–1200 cm^−1^ (Figure 1). This seriously hinders the quantification of individual carbohydrates [32, 33]. Hence, MIR-PLS was proposed as a method for the determination of individual sugars. The C–O stretching band in the spectral region 850–1200 cm^−1^ was selected due to higher sensitivity of the spectra to develop a calibration model [33]. The MIR spectra of selected fermented alcoholic beverages are shown in Figure 2. It can be seen that the MIR spectrum of *Korefe* resembles very much the spectrum of ethanol given in Figure 1. This is because of relatively low sugar content in the *Korefe* compared to the sugar content in the *Birz* and *Tej*.

Two models using sugars (fructose, sucrose, glucose, and maltose) with and without ethanol were used by modifying Rambla et al. [32] and Leopold et al. [33] models. To determine sugars using the MIR-PLS method, a method without ethanol was chosen to avoid interference. To avoid overfitting, the number of principal components (PCs) was fixed to 6 for glucose, fructose, sucrose, and maltose in the model.

### 3.3. Pretreatment Methods

In order to find the model with best prediction capacity, PLS regression was applied to different spectra such as raw spectra, first derivative spectra, second derivative spectra, and others. Leopold et al. [33] also applied PLS regression to raw spectra, first derivative spectra, and second derivative spectra. In their study, lower RMSEP values were provided by the first derivative spectra. But in the present study, the second derivative spectra provided the lower RMSEP values (Table 3). This is because the second derivative preprocessing removes background and increases spectral resolution [34]. Hence, the second derivative spectra were used for the prediction of sugar concentration in the alcoholic beverages.

### 3.4. Method Validation

To avoid concentration data overfitting, the cross-validation method, leaving out one sample at a time, was used. Accordingly, the validation was done using 50 synthetic samples. The obtained validations showed that the PLS calibration model of each has a very good performance (Table 3). It was also found comparable with the report of Irudayaraj and Tewari [35] in terms of correlation (*R*^2^), RMSEP, and the number of factors used. In addition, the predicted amounts were evaluated and compared with the measured values at the 99% confidence level. Overall, no significant variations were obtained between the measured and predicted amounts.

**Figure 3:**
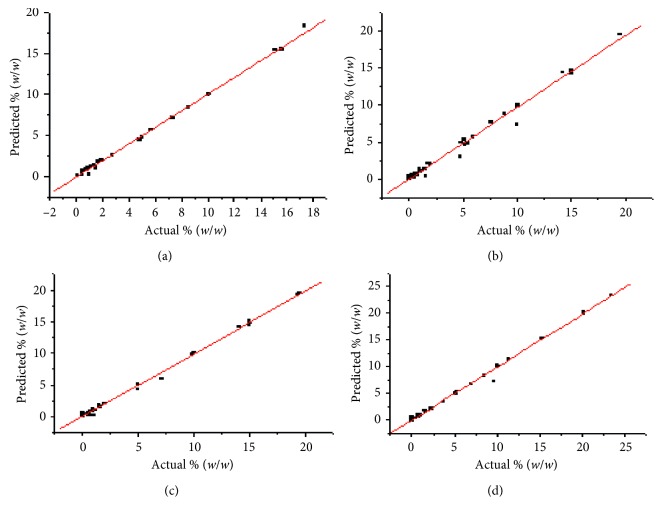
Correlation statistics between actual and predicted values for fructose (a), glucose (b), maltose (c), and sucrose (d).

The correlations between actual and predicted values of the analytes are shown in Figure 3. From the correlation graphs (Figure 3), almost all the curves have shown better correlations. The model was validated in terms of % error using the following equation:

(1) $$\begin{array}{c} \%\;\mathrm{error}=\frac{\mathrm{PLS}\;\mathrm{predicted}\;\mathrm{values}-\mathrm{actual}\;\mathrm{values}}{\mathrm{actual}\;\mathrm{values}}\times 100. \end{array}$$

The % error was found in the range 0.21–3.7% (3.7% glucose, 1.1% fructose, 0.21% sucrose, and 0.23% maltose). This showed that the proposed method has less than 4% error. Thus, the MIR-PLS method was found to give good prediction of sugars in the alcoholic beverages.

### 3.5. Comparisons of MIR-PLS with HPLC-RI

**Figure 4:**
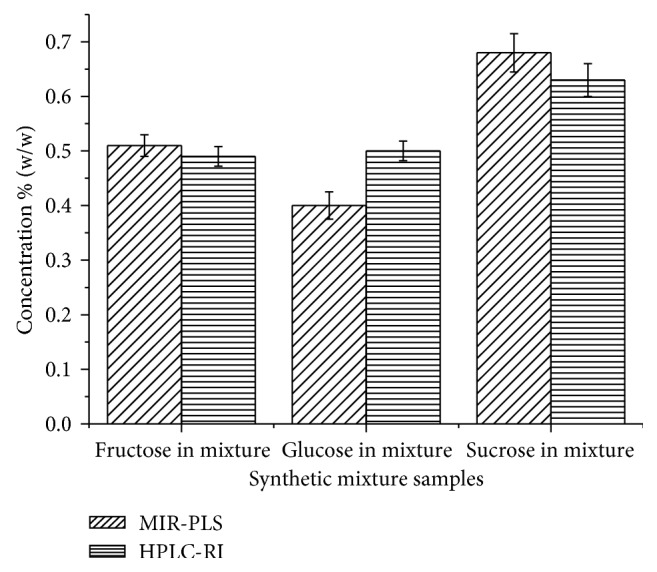
Comparison of MIR-PLS with HPLC-RI.

Comparison of sugar content obtained by MIR-PLS and HPLC-RI was made. The comparison is illustrated in Figure 4. Fructose showed the best comparison, while glucose showed the least comparison among all the sugars. The two techniques were found to be comparable and showed no significance difference at the 95% confidence level. Hence, MIR-PLS was found to be a promising method for sugar determination in the alcoholic beverages. However, to apply for the analysis of real samples, the samples should be purified from potential interferences such as ethanol, phenolic compounds, proteins, amino acids, and others [36, 37].

### 3.6. Determination of Individual Sugars with Derivatization Using HPLC-UV

**Figure 5:**
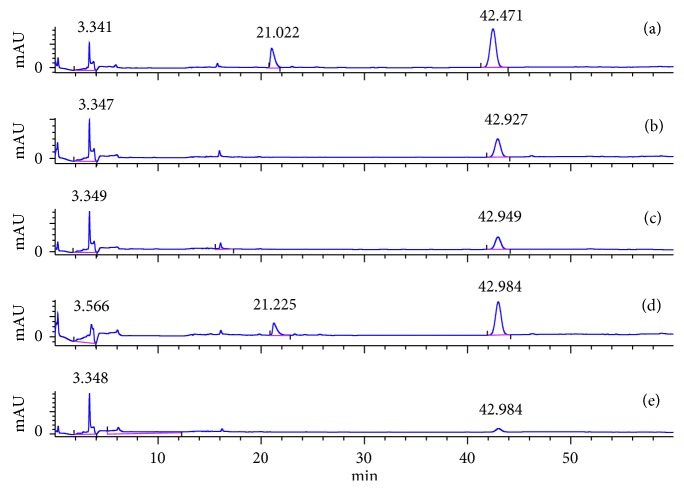
Chromatograms of sugar derivatives: (a) glucose, (b) fructose, (c) sucrose, (d) mixture, and (e) ABEE.

Carbohydrates lack chromophore [11], and they cannot be detected by the UV detector in the HPLC-UV. Thus, they require derivatization. In this study, the derivatization was done using glucose, fructose, and sucrose with *p*-aminobenzoic acid ethyl ester (ABEE), acetic acid, and sodium borohydride. The imine formation was preceded for aldose, not for ketoses and sucrose. Thus, the obtained results are in agreement with different literature reports [1, 31] (Figure 5).

**Scheme 1:**
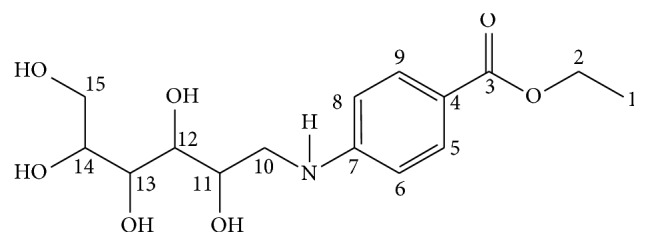
The structure of derivatized glucose.

It can be clearly seen in the chromatogram (Figure 5) that only glucose and mixture have peaks at 21 min retention time due to the glucose labeling, while all the chromatograms (a–e) have peaks at 42 min, owing to excess ABEE. The structure of derivatized glucose (the formed compound) is shown in Scheme 1.

Glucose derivatization was further confirmed by ^1^H NMR and ^13^C NMR analysis. ^1^H NMR spectrum of the compound Glu-ABEE showed the presence of four protons on 1,4-disubstituted aromatic ring appearing at *δ* 6.73 ppm (2H on C_6_ and C_8_; *J* = 8.4 Hz) and 7.69 ppm (2H on C_5_ and C_9_; *J* = 8.4 Hz). This suggested that the four protons are on different chemical environments (i.e., two in one and the remaining two in another environment). In addition, based on the *J* values, they are protons which are coupled. The proton signals between *δ* 3 ppm and 3.6 ppm are due to the protons of OH glucose. The signals at *δ* 4.2 ppm (2H on C_2_; quartet) and *δ* 1.3 ppm (3H on C_1_; triplet) are due to CH_2_ and CH_3_ of the ester, respectively. The signals at *δ* 2.5 ppm are due to DMSO.

The ^13^C NMR analyzed with the DEPT-135 spectrum revealed the presence of three quaternary (from the benzene ring at *δ* 152 ppm (C_7_) and *δ* 118 ppm (C_4_) and from the carboxyl group of ester at *δ* 166 ppm (C_3_)) and three methylene carbon atoms (at *δ* 59.94 ppm (C_10_), *δ* 60.14 ppm (C_15_), and *δ* 60.95 ppm (C_2_)). The signal at *δ* 112 (C_6_ and C_8_) and *δ* 131 (C_5_ and C_9_) was from methine carbons of the ring. The ^13^C NMR signals at *δ* 76 ppm (C_11_), 74.69 ppm (C_12_), 70.42 ppm (C_13_), and 84.98 ppm (C_14_) were from the Cs of the glucose part. The signal at *δ* 14.78 ppm (C_1_) was from the methyl of the ester group. Moreover, the absence of signal around *δ*=100 ppm confirmed that the glucose ring was opened and further reaction occurred. Thus, the ^1^H NMR and ^13^C NMR spectra of the compound confirmed that the derivatization has occurred.

HPLC chromatogram and NMR data confirmed that the labeling is only for glucose. Therefore, only glucose can be determined. To construct a calibration curve, a series of glucose standards from 0.0002 mg/L to 0.002 mg/L were prepared in triplicate. Accordingly, the average calibration equation *y* = 6.41 × 10^5^ *x* – 84 (where *y* = peak area and *x* = glucose in g/mL) with *R*^2^ = 0.9997 was obtained. Therefore, for the determination of glucose, this alternative approach was chosen for two reasons. First, the reducing agent NaBH_4_ is less toxic and easily available than the usual reducing agent NaBH_3_CN, and second, the method has a wider linear range and better correlation.

### 3.7. Determination of Sugars Using HPLC-RI and Sulfuric Acid Method

**Figure 6:**
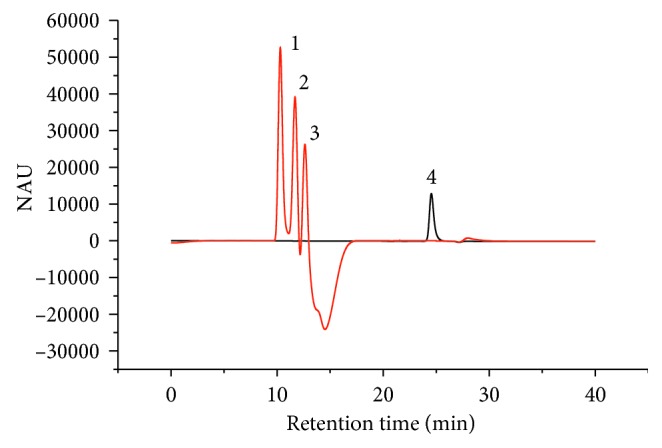
Chromatograms of the mixture of sugars and ethanol: 1 = sucrose, 2 = glucose, 3 = fructose, and 4 = ethanol. Column: Hi-Plex H 7.7 × 300 mm ID (35°C). Mobile phase: water. Flow rate: 0.5 mL/min. Injection volume: 10 *µ*L.

The chromatograms of sugars and ethanol are illustrated in Figure 6. For the determination of sugars in the real samples, peak identifications were made in the same chromatographic system by comparing each peak's retention time (*t*_R_) with the reference (standards) and by spiking the sample with the standards. Standards solutions of 0.03–0.2% (w/w) for glucose, fructose, and sucrose and standards solution of 0.01–0.1 g/L for the total sugars were prepared in triplicate. The calibration equations *y* = 1.87 × 10^6^ *x* + 1012, *y* = 2.02 × 10^6^ *x* – 1836, and *y* = 2.00 *x* + 2241 (where *y* = peak area and *x* = concentration of analytes in % (w/w)) were assigned for fructose, glucose, and sucrose, respectively. The calibration equation used for the total sugars was *y* = 10.6 *x* – 0.022 (where *y* = peak height and *x* = glucose concentration in g/L).

### 3.8. Method Validation

The proposed method was validated using the recovery test. For recovery determination of fructose, glucose, and sucrose, 0.05 and 0.1% (w/w) from each type were spiked. But for the recovery determination of total sugars, 6.5 and 13.0 g/L glucose were spiked. The results are presented in Table 4. LOD = 3*σ* of the residues (*y*-intercepts)/slope [38] was also determined and given in Table 4. The recovery percentages obtained for fructose (89–106%), glucose (92–109%), sucrose (94–95%), and total carbohydrate (98–109%) are in the acceptable ranges. This implies that the matrix effects of the samples were not considerable. Therefore, the proposed techniques are appropriate to quantify individual and total sugar contents in the fermented alcoholic beverages.

### 3.9. Comparison of Advantages and Disadvantages of the Four Methods

In this study, four methods (MIR-PLS, HPLC-UV, HPLC-RI, and sulfuric acid methods) have been developed for the determination of sugars in the fermented alcoholic beverages. A comparison has been made on the advantages and disadvantages of the individual methods, and the results are summarized in Table 5. The MIR-PLS method is applicable to determine individual sugars: glucose, fructose, sucrose, and maltose, with good precision and accuracy. But the method suffers from interferences from ethanol, phenolic compounds, proteins, amino acids, and others. The HPLC-UV method is only applicable to determine glucose but not other individual sugars. It also requires derivatization which is time-consuming. The HPLC-RI method is applicable to determine individual sugars: glucose, fructose, and sucrose, without the matrix effect but not other individual sugars. The sulfuric acid method is applicable to determine total sugars with good precision and accuracy. However the method is not applicable to determine individual sugars.

### 3.10. Analysis of Real Samples

The amounts of sugars found in the real samples (fermented alcoholic beverages) are presented in Table 6. The amounts of individual sugars are expressed as % (w/w) and the total sugar as g/L in Ethiopian traditional fermented alcoholic beverages. This is because in the literature, the individual sugar contents are expressed as % (w/w) and the total sugar content as g/L to make the comparison of results of the present study with the results reported in the literature meaningful. The order of the beverages based on the total sugar contents (in g/L) was *Birz* > *Tej* > *Keribo* > *Korefe* > *Netch Tella* > *Filter Tella* > *Tella* > *Borde*. *Birz* and *Tej* were found to be the leading ones. Particularly for *Birz*, it is mainly by the unfermented sugars left after fermentation. In *Tej*, additional honey was added, while it is ready to serve. For the rest of the beverage types, the major reason of variation is the differences in the composition of the raw materials (Table 1) and fermentation time used [25, 30].

Although it is not reasonable to compare beverages of different types, the comparisons were done without considering the factors that can influence their levels of sugars. In this study, *Birz* was the first in the levels of glucose and fructose, while *Keribo* was the first in the level of sucrose. Both controlled *Tella* and *Tej* have comparable glucose, fructose, and sucrose with the average values of the corresponding samples. However, there were still variations among beverages. This showed that the variation in the composition of the raw materials, preparation process, and fermentation time used (Table 1) are the crucial factors for the difference in the level of sugars in the traditional fermented beverages.

The total sugar contents with average values of 37.0 g/L in nonalcoholic beer [39], 42.1 g/L in alcoholic beer [39], 90.2 g/L in wort from phases I–V [40], and 6.75 g/L [41] and 48.8 g/L [42] in wine were reported. The Ethiopian traditional fermented alcoholic beverages have been found to contain less sugar than in wort [40] and beer [39] except *Birz*, *Tej*, and *Korefe.* But they have higher sugar than that reported by Matsuhiro et al. [41].

In some beverages, the concentration of glucose, fructose, and sucrose was reported. The concentration values of sucrose, glucose, and fructose were found in the ranges: 1–3.28 and 0–10.5, 0.13–2.17 and 0–6.9, and 0.3–3.72 and 0–6.7% (w/w), respectively, in flavored water and soft drinks, respectively [43, 44]. Since both the flavored water and soft drinks are not fermented beverages, their sugar contents are expected to be higher than those in fermented beverages. Even though the Ethiopian traditional fermented beverages have passed through some extent of fermentation, the obtained results confirmed that the Ethiopian fermented alcoholic beverages have comparable sugar content with that in the nonfermented beverages.

## 4. Conclusion

Improvement in the performance of analytical methods for the determination of sugars in alcoholic beverages using MIR-PLS, HPLC-UV with a derivatizing agent, and HPLC-RI has been presented. The MIR-PLS method is applicable to determine individual sugars: glucose, fructose, sucrose, and maltose, with good precision and accuracy. The HPLC-RI method is applicable to determine individual sugars: glucose, fructose, and sucrose, without the matrix effect. The HPLC-UV method is applicable to determine only glucose but not other sugars. The sulfuric acid method is applicable to determine total sugar with good precision and accuracy. The amount of individual sugars in Ethiopian traditional fermented alcoholic beverages was determined by using the HPLC-RI method, and the total sugar was determined by using the sulfuric acid method. The sugar contents in the Ethiopian fermented alcoholic beverages are comparable with those in the commercial beverages. The differences in the level of sugars in the different types of traditional fermented beverages are due to the variation in the composition of the raw materials, preparation process, and fermentation time used in the traditional fermented beverages.

## Figures and Tables

**Table 1:** Physicochemical properties, raw materials, and production process of some Ethiopian traditional alcoholic beverages.

S. no.	Samples (% alcohol)^*∗*^	Physicochemical properties	Raw materials	Production process
1	*Tella* (2.9 ± 0.3), *Korefe* (4.6 ± 0.4)	Dark brown in color, with pH 3.56 ± 0.02 and 4.28 ± 0.02	*Kita* (a thin, 5–10 mm thick, pancake-like bread), *enkuro* (a dark brown toasted flour), *bikil* (germinated grain), and powdered *gesho* (*Rhamnus prinoides*) [25, 26]	A four-phase fermentation for 10–12 days [25, 26]
2	*Tej* (9.1 ± 0.3)	Yellow, sweet, effervescent, and cloudy, with pH 3.65 ± 0.01	Honey or mixture of sugar with honey and leaves of *gesho* (*Rhamnus prinoides*) [25, 27]	Mixing boiled must with *gesho* (*Rhamnus prinoides*) and unboiled must and then allowing to ferment for 5 days in warm weather or for 15–20 days in colder weather [25, 27]
3	*Birz* (6.5 ± 0.8)	Yellow, sweet, effervescent, and cloudy, with pH 3.40 ± 0.06	Honey or mixture of sugar with honey [25]	Has a short fermentation period, usually overnight [25]
4	*Borde* (1.8 ± 0.4)	Opaque, effervescent, and whitish-grey to brown colored with a thick consistency and a sweet-sour taste, with pH 5.77 ± 0.03	Unmalted maize (*Zea mays*), barley (*Hordeum vulgare*), wheat (*Triticum aestivum*), finger millet (*Eleusine coracana*), sorghum (*Sorghum bicolor*), and/or *tef* (*Eragrostis tef*) and their malt; additional ingredients garlic, fresh chili (*Capsicum minimum*), ginger, and salt [25, 28, 29]	A four-phase fermentation for less than 4 days [25, 28, 29]
5	*Keribo* (1.7 ± 0.3)	Dark brown colored, with pH 3.72 ± 0.03	Unmalted roasted barley (*Hordeum vulgare*), sugar, and yeast [30]	Has a short fermentation period, usually overnight [30]

^*∗*^% alcohol was determined by the method in [22].

**Table 2:** Optimized extraction time for sugar extraction from semiliquid samples.

Extraction time (min)	30	60	90	120
Total carbohydrate (Glu·(g/L))	14.9 ± 0.1	15.1 ± 0.1	25.8 ± 0.2	13.4 ± 0.2

**Table 3:** Results of MIR-PLS calibration models for the determination of sugars in alcoholic beverages.

Data treatment	Analytes	Principal components	Calibration		Validation	
			*R* ^2^	RMSEE	*R* ^2^	RMSEP
Second derivative	Glucose	6	0.992	0.18	0.987	0.55
Second derivative	Fructose	6	0.997	0.06	0.996	0.28
Second derivative	Sucrose	6	0.989	0.22	0.996	0.38
Second derivative	Maltose	6	0.999	0.04	0.997	0.29

RMSEE: root mean square error of estimation; RMSEP: root mean square error of prediction.

**Table 4:** Limit of detection and recovery percentage for individual sugars by the HPLC-RI method and for total sugar by the sulfuric acid method.

S. no.	Sample	Fructose		Glucose		Sucrose		Total sugar	
		LOD (%, w/w)	Recovery (%)	LOD (%, w/w)	Recovery (%)	LOD (%, w/w)	Recovery (%)	LOD (g/L)	Recovery (%)
1	*Keribo*	0.001	106	0.002	93.2	0.002	94.3	0.009	98.2
2	*Tej*	0.001	89.4	0.002	109	0.002	94.2	0.009	100
3	*Tella*	0.001	90.3	0.002	92.4	0.002	95.1	0.009	109

**Table 5:** Comparison of advantages and disadvantages of the four newly developed methods for the determination of sugars in the fermented alcoholic beverages.

Method	Advantages	Disadvantages
MIR-PLS method	The method is applicable to determine individual sugars: glucose, fructose, sucrose, and maltose, with good precision and accuracy.	To apply for the analysis of real samples, the samples should be purified from potential interferences such as ethanol, phenolic compounds, proteins, amino acids, and others.
HPLC-UV method	The method is applicable to determine glucose, and it has a wider linear range and better correlation.	The method is not applicable to determine individual sugars except glucose, and it requires derivatization which is time-consuming.
HPLC-RI method	The method is applicable to determine individual sugars: glucose, fructose, and sucrose, without the matrix effect.	The method is applicable to determine individual sugars: glucose, fructose, and sucrose, but not other sugars.
Sulfuric acid method	The method is applicable to determine total sugar (carbohydrate) with good precision and accuracy.	The method is not applicable to determine individual sugars.

**Table 6:** The amount of sugars found in Ethiopian traditional fermented alcoholic beverages (individual sugars (%, w/w) by the HPLC-RI method and total sugar (g/L) by the sulfuric acid method).

S. no.	Beverage types	Number	Concentration of individual sugars (%, w/w)			Total carbohydrate (g/L)
			Fructose	Glucose	Sucrose and maltose	
1	*Tella*	15	0.09 ± 0.12	0.07 ± 0.09	0.08 ± 0.12	12.0 ± 4.9
2	*Tej*	15	2.98 ± 1.10	1.66 ± 0.60	0.19 ± 0.20	64.5 ± 24
3	*Korefe*	6	0.30 ± 0.10	0.43 ± 0.30	0.18 ± 0.15	34.4 ± 13.5
4	*Keribo*	6	0.10 ± 0.05	0.60 ± 0.03	3.00 ± 0.30	50.0 ± 9.1
5	*Birz*	4	8.50 ± 0.50	5.60 ± 1.40	1.00 ± 0.70	131 ± 18
6	*Borde*	3	0.09 ± 0.01	4.33 ± 0.01	0.40 ± 0.01	11.3 ± 1.25
7	*Netch Tella*	4	0.70 ± 1.33	0.79 ± 1.35	0.08 ± 0.03	23.3 ± 2.8
8	*Filter Tella*	4	1.40 ± 2.70	0.90 ± 1.60	0.36 ± 0.60	19.0 ± 4.1
9	Control *Tella*	3	0.04 ± 0.01	0.03 ± 0.01	0.12 ± 0.01	102 ± 1.8
10	Control *Tej*	3	1.77 ± 0.05	1.11 ± 0.04	0.14 ± 0.01	270 ± 5.7

## Data Availability

The data used to support the findings of this study are available from the corresponding author upon request.
